# Positive LGI1 Antibodies in CSF and Relapse Relate to Worse Outcome in Anti-LGI1 Encephalitis

**DOI:** 10.3389/fimmu.2021.772096

**Published:** 2021-12-17

**Authors:** Li-li Cui, Johannes Boltze, Yan Zhang

**Affiliations:** ^1^ Department of Neurology, Xuanwu Hospital, Capital Medical University, Beijing, China; ^2^ School of Life Sciences, University of Warwick, Coventry, United Kingdom; ^3^ Institute of Sleep and Consciousness Disorders, Beijing Institute of Brain Disorders, Collaborative Innovation Center for Brain Disorders, Capital Medical University, Beijing, China

**Keywords:** anti-LGI1 encephalitis, outcome, CSF antibody, relapse, risk factor

## Abstract

**Objective:**

This single-center study was conducted in a cohort of patients with anti-LGI1 encephalitis to investigate the factors related to their functional recovery.

**Methods:**

We retrospectively collected the clinical information of patients admitted to Xuanwu Hospital from January 2014 until December 2019, and followed up for at least 12 months.

**Results:**

A total of 67 patients were included, and 57 completed the 12-month follow-up. Most of the patients (55/57, 96.5%) achieved functional improvement after immunotherapy, and 26 (45.6%) became symptom-free. Compared to patients with complete recovery, patients with partial or no recovery had significantly higher incidences of consciousness disorders (25.8% vs. 0%, P<0.05) and positive LGI1 antibodies in cerebrospinal fluid (CSF) (71.0% vs. 46.2%, P<0.05). These patients also had a lower Barthel Index both upon admission and at discharge, as well as a higher incidence of relapse (25.8% vs. 3.8%; P<0.05 each). Univariate logistic regression showed that positive LGI1 antibodies in CSF and relapse were associated with incomplete recovery at 1-year follow-up (both P<0.05), but only relapse remained statistically significant after multivariate logistic regression (P=0.034).

**Conclusion:**

Patients with LGI1 antibodies in CSF and those who relapsed were more likely to experience worse outcome. Early recognition of these patients, combined with more aggressive immunotherapy may result in better recovery.

## Introduction

Anti-LGI1 (leucine-rich glioma-inactivated 1) encephalitis is a newly discovered autoimmune encephalitis and a relatively common cause of limbic encephalitis (1). Recent publications reported the key symptoms of anti-LGI1 encephalitis patients, including subacute cognitive decline and seizures (particularly faciobrachial dystonic seizures), as well as a good response to immunotherapy (1–5). Long-term outcome has been described in some studies (2–5), but little is known about the potential risk factors of unfavorable outcome or relapse. This study aimed to explore these factors.

## Methods

### Patients

We retrospectively identified patients with definite anti-LGI1 encephalitis admitted to the Department of Neurology of Xuanwu Hospital, Capital Medical University, between January 2014 and December 2019 (**Supplemental Figure 1**). The diagnosis was made when the patient met all of the following criteria: 1) presence of clinical symptoms involving the limbic system (subacute onset of working memory deficits, seizures, or psychiatric symptoms); 2) abnormalities of the medial temporal lobes on T2-weighted fluid-attenuated inversion recovery (T2-FLAIR) magnetic resonance imaging (MRI), or CSF pleocytosis, or electroencephalogram (EEG) with epileptic or slow-wave activity involving the temporal lobes; 3) detection of anti-LGI1 antibody in serum and/or cerebrospinal fluid (CSF); 4) exclusion of alternative causes (6).

The LGI1 antibodies in both serum and CSF were detected using indirect immunofluorescence test (IIFT) kits with transfected cells (EUROIMMUN, Lübeck, Germany, Cat.No. FA 112d-1005-13) following the manufacturer’s instructions. Briefly, the slides were incubated with serum samples starting from a dilution of 1:10 while CSF samples undiluted. The positivity of anti-LGI1 antibody was defined as antibody titer semiquantitatively using serial dilution of serum and CSF samples, with the titer being the last dilution that showed visible reactivity.

Clinical information was obtained from medical records or by interviewing patients and their relatives on site or by telephone. Patients were followed up for at least 12 months after discharge. The Barthel Index was used to assess the patients’ abilities of daily living both upon admission and at discharge, with 0 being full assistance needed and 100 being totally independent (**Supplemental Table 1**). The modified Ranking Scale (mRS) was used to evaluate functional outcome, with 0 considered as complete recovery and 6 as death (**Supplemental Table 2**). Relapse was defined as clinical worsening during treatment after initial improvement that required a change of medication, or clinical worsening observed after recovery and being off-medication.

### Standard Protocol Approvals, Registrations, and Patient Consents

This study was approved by the Ethics Committee of Xuanwu Hospital, Capital Medical University (No. 2020-059) and complied with the Declaration of Helsinki. Informed consents were obtained from patients or their relatives.

### Statistical Analysis

Statistical analyses were performed using SPSS 22.0 (IBM Corporation, Armonk, NY). Data are presented as mean ± standard deviation (SD), median (interquartile range, IQR) or count (percentages), and were compared using Student’s t-test, Mann-Whitney U-test, or chi-square test when appropriate. Relationships between clinical characteristics and functional outcome at 12 months or relapse were explored by logistic regression or Spearman’s correlation analysis. Receiver operating characteristic (ROC) curve analysis was performed to explore the cutoff value to predict relapse. All analyses were 2-tailed, and P<0.05 was considered statistically significant.

### Data Availability

Any data not published within the article are available and will be shared upon reasonable request from any qualified investigator.

## Results

### Patient Characteristics

**Figure 1:**
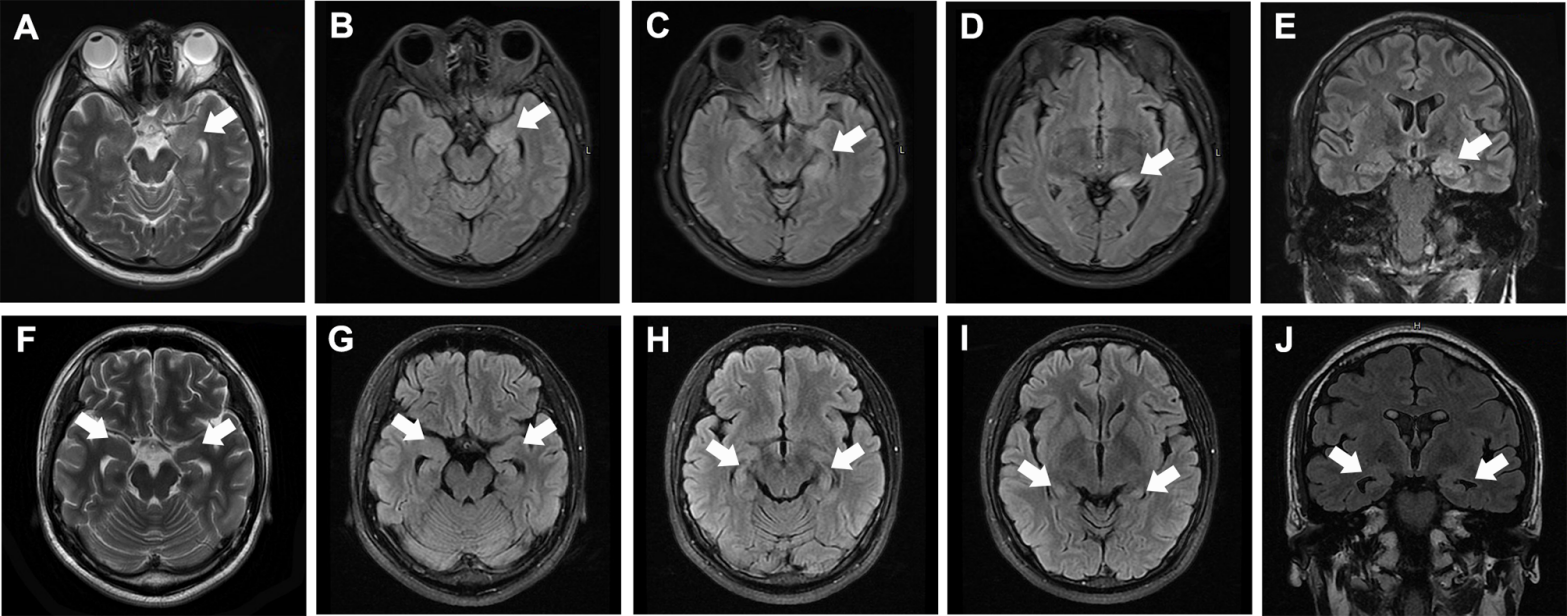
Typical MRI of anti-LGI1 encephalitis patients. **(A–E)** MRI of a patient at 1 month after disease-onset, showing high T2 **(A)** and T2/FLAIR [**(B–D** axial, **(E)** coronal] signals in the left hippocampus (white arrows). **(F–J)** MRI of a patient at 6 months after disease-onset, showing moderately elevated T2 **(A)** and T2/FLAIR [**(B–D)** axial, **(E)** coronal] signals in both hippocampi (white arrows) as well as bilateral hippocampal atrophy.

**Figure 2:**
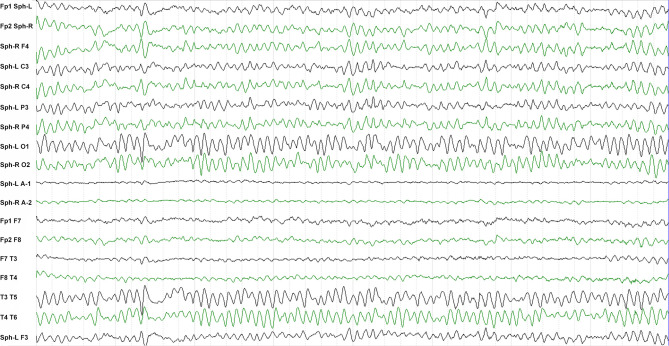
Typical EEG of a patient with anti-LGI1 encephalitis. The inter-ictal EEG of a patient shows sharp-wave activity in the sphenoid electrodes.

Sixty-seven patients (38 males, 29 females, average age 58.5 ± 13.9 years) diagnosed with anti-LGI1 encephalitis were enrolled (**Table 1**). The most frequent initial symptoms were seizures (89.6%), cognitive impairment (70.1%), and psychosis (31.3%). LGI1 antibodies were detected in CSF of 42 (62.7%) patients and in serum samples of all patients. Abnormal MRI and EEG findings were detected in 32 (47.7%) and 33 (49.3%) patients, respectively (see the typical MRI and EEG findings in **Figure 1** and **Figure 2**, respectively). The median of mRS upon admission was 2 (IQR: 1~3).

**Table 1:** Patient characteristics (N = 67).

Characteristics	Values
**Male/Female**	38/29
**Age at onset, y, mean ± SD**	58.5 ± 13.9
**Symptom at diagnosis, n (%)**	
**Seizure**	60 (89.6%)
**Cognitive impairment**	47 (70.1%)
**Psychosis**	21 (31.3%)
**Consciousness disorder**	9 (13.4%)
**Speech disorder**	4 (6.0%)
**Sleep disorder**	4 (6.0%)
**Autonomic dysfunction**	6 (9.0%)
**Limb weakness**	6 (9.0%)
**Hyponatremia, n (%)**	29 (43.3%)
**CSF findings, n (%)**	
**Cell count > 5 cells/uL**	10 (14.9%)
**Protein > 0.45 g/L**	17 (25.4%)
**Positive OB**	7 (10.4%)
**LGI1 antibody, n (%)**	
**CSF positive**	42 (62.7%)
**Serum positive**	67 (100%)
**EEG, n (%)**	
**Normal**	14 (20.9%)
**Epileptic**	24 (35.8%)
**Slow wave**	8 (11.9%)
**Unclear**	21 (31.4%)
**MRI, n (%)**	
**Normal**	34 (50.7%)
**Unilateral hippocampal lesion**	16 (23.9%)
**Bilateral hippocampal lesions**	17 (25.4%)
**Tumor, n (%)**	6 (9.0%)
**BI upon admission, mean ± SD**	80.3 ± 18.4
**BI at discharge, mean ± SD**	82.5 ± 20.8
**mRS upon admission, median (IQR)**	2 (1~3)

SD, standard deviation; CSF, cerebrospinal fluid; OB, oligoclonal band; EEG, electroencephalography; MRI, magnetic resonance imaging; BI, Barthel Index; mRS, modified Ranking scale; IQR, interquartile range.

### Functional Outcome at 12 Months

**Figure 3:**
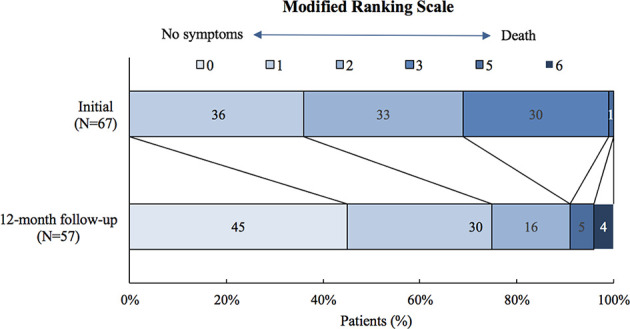
Modified ranking scale (mRS) of patients with anti-LGI1encephalitis at the 12-month follow-up.

Fifty-seven patients completed the 12-month follow-up. Their mRS decreased to a median of 1 (IQR: 0~1) at 12 months (**Figure 3**). Most of the patients (55/57, 96.5%) achieved functional improvement after treatment; 26 (45.6%) patients became symptom-free. The most common residual symptoms were cognitive impairment (51.9%), followed by epileptic seizures (22.2%) and personality changes (21.6%, particularly irritability).

Compared to patients with complete recovery, those experiencing partial or no recovery had a significantly higher incidence of consciousness disorder (25.8% vs. 0.0%), and were more often CSF-positive for LGI1 antibodies (71.0% vs. 46.2%; P<0.05 each). They more frequently received intravenous immunoglobin (IVIG) (64.5% vs. 34.6%), had a higher incidence of relapse (25.8% vs. 3.8%), and a significantly lower Barthel Index both upon admission and at discharge (P<0.05 each, **Table 2**). Univariate logistic regression revealed that LGI1 antibodies in CSF [odds ratio (OR)=3.208, 95% CI: 1.049-9.811, P=0.041] and relapse (OR=9.091, 95% CI: 1.052-78.543, P=0.045) were associated with incomplete recovery, while only relapse remained statistically significant after multivariate logistic regression (OR=11.047, 95% CI: 1.196-102.064, P=0.034).

**Table 2:** Comparison between patients with complete recovery and those experiencing incomplete or no recovery at 12 months after discharge.

	Complete recovery (mRS = 0) N = 26	Incomplete or no recovery (mRS > 0) N = 31	P value
**Male/Female**	12/14	18/13	0.37
**Age at onset, y, mean ± SD**	54.8 ± 13.7	60.1 ± 14.7	0.17
**Symptom at diagnosis, n (%)**			
**Seizure**	22 (84.6%)	29 (93.5%)	0.396
**Cognitive impairment**	17 (65.4%)	23 (74.2%)	0.469
**Psychosis**	6 (24.0%)	10 (32.3%)	0.496
**Consciousness disorder**	0	8 (25.8%)	0.006^**^
**Speech disorder**	2 (8.3%)	1 (3.2%)	0.575
**Sleep disorder**	1 (3.8%)	2 (6.5%)	1.0
**Autonomic dysfunction**	3 (11.5%)	2 (6.5%)	0.837
**Limb weakness**	2 (7.7%)	2 (6.5%)	1.0
**Hyponatremia, n (%)**	10 (38.5%)	14 (45.2%)	0.61
**CSF findings, n (%)**			
**Cell count > 5 cells/uL**	4 (15.4%)	5 (16.1%)	1.0
**Protein > 0.45 g/L**	6 (24.0%)	7 (22.6%)	0.991
**Positive OB**	2 (8.3%)	3 (9.7%)	1.0
**LGI1 antibody, n (%)**			
**CSF positive**	12 (46.2%)	22 (71.0%)	0.038^*^
**Serum positive**	25 (100%)	27 (100%)	
**EEG, n (%)**			
**Normal**	7 (26.9%)	3 (9.7%)	
**Epileptic discharge**	10 (38.5%)	9 (29.0%)	
**Slow wave**	1 (3.8%)	5 (16.1%)	0.054
**MRI, n (%)**			
**Normal**	11 (42.3%)	13 (41.9%)	
**Unilateral hippocampal lesion**	5 (19.2%)	8 (25.8%)	
**Bilateral hippocampal lesions**	6 (23.1%)	7 (22.6%)	
**Others**	4 (15.4%)	1 (3.2%)	0.461
**Tumor, n (%)**	3 (11.5%)	2 (6.5%)	0.233
**Initial treatment, n (%)**			
**Corticosteroid**	21 (80.8%)	24 (77.4%)	0.942
**IVIG**	9 (34.6%)	20 (64.5%)	0.017^*^
**MMF**	1 (3.8%)	5 (16.1%)	0.2
**Antiepileptic drugs**	22 (84.6%)	26 (83.9%)	1.0
**BI upon admission, mean ± SD**	86.0 ± 14.5	76.0 ± 20.3	0.04^*^
**BI at discharge, mean ± SD**	89.2 ± 13.2	79.4 ± 20.9	0.042^*^
**Relapse, n (%)**	1 (3.8%)	8 (25.8%)	0.029^*^
**mRS upon admission, median (IQR)**	2 (1~2)	2 (1~3)	0.053

SD, standard deviation; CSF, cerebrospinal fluid; OB, oligoclonal band; EEG, electroencephalography; MRI, magnetic resonance imaging; IVIG, intravenous immunoglobins; MMF, mycophenolate mofetil; BI, Barthel Index; mRS, modified Ranking scale; IQR, interquartile range. ^*^P < 0.05; ^**^P < 0.01.

Bold values indicate statistical significance.

### LGI1 Antibodies in CSF

Patients with positive CSF LGI1 antibodies were significantly older (61.2 ± 12.5 vs. 53.6 ± 15.0 years, P=0.034), were more likely to show cognitive deficits (78.6% vs. 52.2%, P=0.027), and had a lower Barthel Index upon admission (76.9 ± 18.4 vs. 87.0 ± 17.4, P=0.036) and at discharge (78.8 ± 23.6 vs. 90.0 ± 13.1, P=0.016). They also exhibited a higher mRS upon admission [2 (1~3) vs. 1 (1~1), P=0.003] and at 12 months’ follow-up [1 (0~2) vs 0 (0~1), P=0.023] (**Supplemental Table 3**).

### Relapse

Ten patients relapsed during the follow-up; 9 of these relapses occurred within 12 months after initial treatment. The relapsing patients showed a higher incidence of residual epileptic seizures (60.0% vs. 12.8%, P<0.05), and tended to be older (65.9 ± 12.3 vs. 56.5 ± 14.2 years, P=0.056), with a lower Barthel Index at discharge (72.5 ± 24.0 vs. 85.2 ± 17.6, P=0.057; **Supplemental Table 4**). They also received more intensive immunotherapy after relapse (**Supplemental Figure 2**).

**Figure 4:**
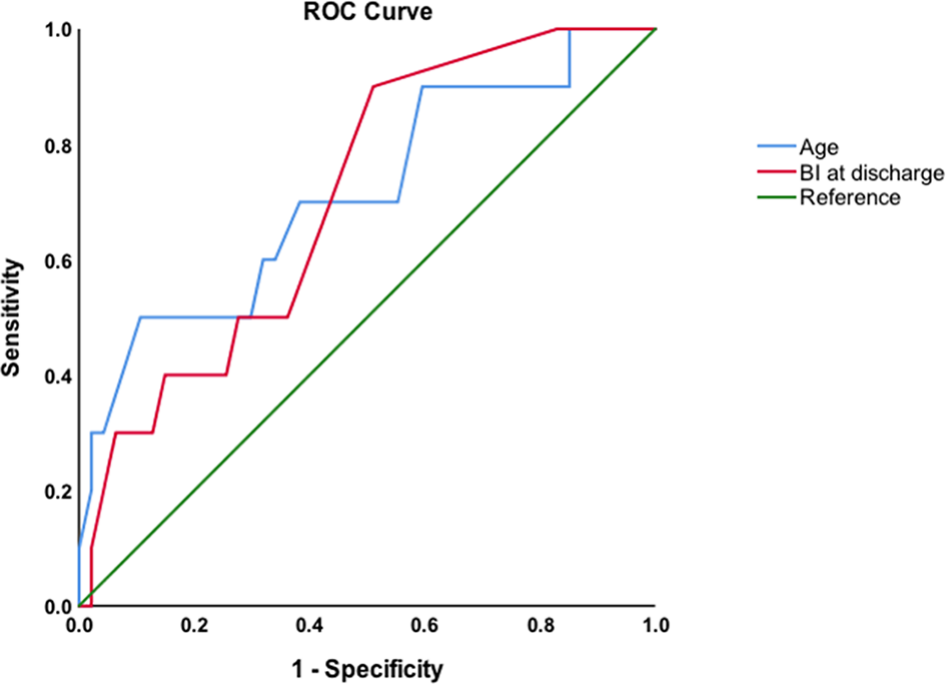
ROC curves of age and Barthel Index (BI) at discharge to predict relapse.

Spearman correlation analysis revealed significant relationships between age, Barthel Index at discharge, and relapse (ρ=0.283 and ρ=-0.278, respectively, P<0.05). The area under the ROC curve of age to predict relapse was 0.715 (P=0.034), with a cutoff value of 70 years old (sensitivity: 50%, specificity: 89.4%). The area under the ROC curve of Barthel Index at discharge to predict relapse was 0.707 (P=0.041), with a cutoff value of 92 (sensitivity: 48.9%, specificity: 90%; **Figure 4**).

## Discussion

Clinical characteristics and potential mechanisms of anti-LGI1 encephalitis still need further elucidation. We investigated the factors associated with unfavorable functional outcome in anti-LGI1 encephalitis, and found that patients with positive CSF LGI1 antibodies and those who relapsed had worse outcome after treatment.

As a neuron-secreted protein, LGI1 forms a trans-synaptic complex with presynaptic proteins ADAM11/23 and postsynaptic proteins ADAM22. LGI1 is involved in synaptic transmission of neuronal excitability, regulating the presynaptic voltage-gated potassium channel complex (VGKC) and postsynaptic α-amino-3-hydroxy-5-methyl-4-isoxazolepropionic acid (AMPA) receptors (1). Consistent with previous studies (2–5), our patients showed typical manifestations such as seizures (particularly FBDS) and cognitive deficit, and some patients had hippocampal T2/FLAIR hyperintense signals or hippocampal atrophy/sclerosis. This is because LGI1 antibodies [mainly of IgG4 subclass (2, 7, 8)] are believed to alter K_v_1.1 subunit of VGKC as well as AMPA receptors, causing neuronal hyperexcitability and reducing long-term synaptic plasticity in the hippocampus (1, 9). HLA class II was reported to relate with anti-LGI1 encephalitis, particularly HLA-DRB1*07:01 (8, 10). However, the exact mechanisms that underlie the effects of anti-LGI1 antibodies remain to be investigated.

Evidence of intrathecal LGI1 antibody synthesis is increasing (7, 11, 12), although passive diffusion from blood cannot be excluded. The positive rate of LGI1 antibody was higher in serum than in CSF in our patients, which is in line with most other studies (3–5, 13, 14) except for that of Ariño et al. (2). However, our patients had a relatively lower detection rate of LGI1 antibodies in CSF and better functional outcome compared to some other studies (2–4). Consistent with our findings, positive LGI1 antibodies in CSF were found to correlate with increased neuronal excitability (11) and poor outcome recently (8). Similarly, an elevated LGI1-IgG CSF index was reported to predict unfavorable neurological outcome (7), indicating that intrathecal antibody synthesis may lead to more severe neuronal impairment. Moreover, being negative for CSF LGI1 antibodies may not exclude intrathecal LGI1 autoantibody production (11), since the antibodies could rapidly bind to their antigens, which may lead to a false-negative CSF diagnosis. Nevertheless, consistent with the findings of Ariño et al. (2), neither LGI1 antibody titers in CSF nor those in serum were found to relate with outcome in our study.

Clinical relapse occurred in 15% of our patients, which is lower than previously reported (2, 3). This was probably related to better recognition of this disease and more timely treatment in recent years. Relapse has been reported to correlate with unfavorable outcome in previous studies (2), but predictors of relapse have not been elucidated. It appears logical that aged patients with a low Barthel Index may have a higher risk of relapse, perhaps due to their impaired immunity and more severe initial symptoms. However, most of the relapsing patients were just treated with steroids or IVIG before relapse, only a few received both treatments. None of them were administered mycophenolate mofetil (MMF) before relapse, but half of the relapsing patients received MMF. Thus, optimal form and duration of immunotherapy for anti-LGI1 encephalitis patients remain to be identified.

A major limitation of this study is that only a cell-based assay (CBA) was performed to detect LGI1 antibodies using commercial kits since the positive rate of LGI1 antibodies in CSF may vary depending on the detection assays (2–5). Although both the sensitivity and specificity of the commercial CBA kit used in this study is claimed as 100%, there has been literature reporting false-negative results using this kit, and a combination of both tissue- and cell-based assays is recommended (3). Another limitation is its retrospective nature with limited availability of blood/CSF samples. Thus some tests (e.g. IgG subclasses of LGI1 antibody and HLA typing) cannot be performed. Others include the relatively small sample size and short follow-up, and the use of only mRS for outcome evaluation. More patients are being prospectively enrolled and a longer follow-up is being performed to monitor their long-term outcome.

## Conclusion

Patients with positive LGI1 antibodies in CSF and those who relapsed were more likely to suffer worse outcome. Early recognition of these patients and providing more aggressive immunotherapy may result in better recovery. Prospective studies with larger sample sizes are warranted to provide more evidence and to enable optimal treatment decisions for these patients.

## Data Availability Statement

The original contributions presented in the study are included in the article/**Supplementary Material**. Further inquiries can be directed to the corresponding author.

## Ethics Statement

The studies involving human participants were reviewed and approved by the Ethics Committee of Xuanwu Hospital, Capital Medical University. The patients/participants provided their written informed consent to participate in this study.

## Author Contributions

LC and YZ contributed to data acquisition and statistical analysis. LC drafted the manuscript. JB contributed to data interpretation and manuscript revision. YZ contributed to the conception and design of the study and manuscript revision. All authors contributed to the article and approved the submitted version.

## Funding

This project was supported by the National Key Research and Development Program of China (2020YFC2005403) and the Beijing Municipal Administration of Hospitals Incubating Program (PX2020035).

## Conflict of Interest

The authors declare that the research was conducted in the absence of any commercial or financial relationships that could be construed as a potential conflict of interest.

## Publisher’s Note

All claims expressed in this article are solely those of the authors and do not necessarily represent those of their affiliated organizations, or those of the publisher, the editors and the reviewers. Any product that may be evaluated in this article, or claim that may be made by its manufacturer, is not guaranteed or endorsed by the publisher.
