# Supplementary figures and images for: Positive LGI1 Antibodies in CSF and Relapse Relate to Worse Outcome in Anti-LGI1 Encephalitis
Source: Front Immunol. 2021 Dec 17;12:772096. doi: 10.3389/fimmu.2021.772096 (PMC8718904; doi:10.3389/fimmu.2021.772096)

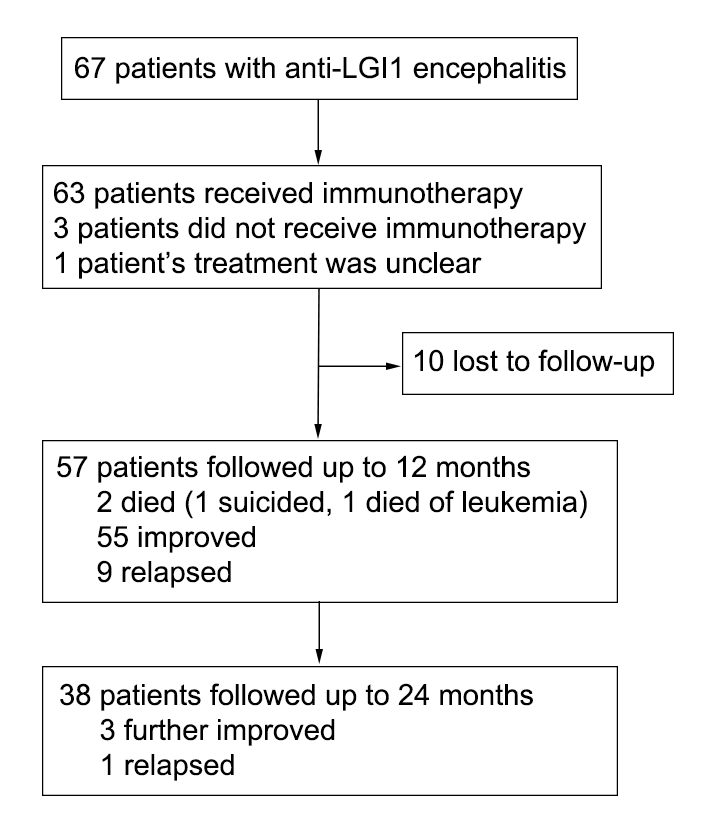

Supplement: Supplementary file 1 [file Image_1.tif]

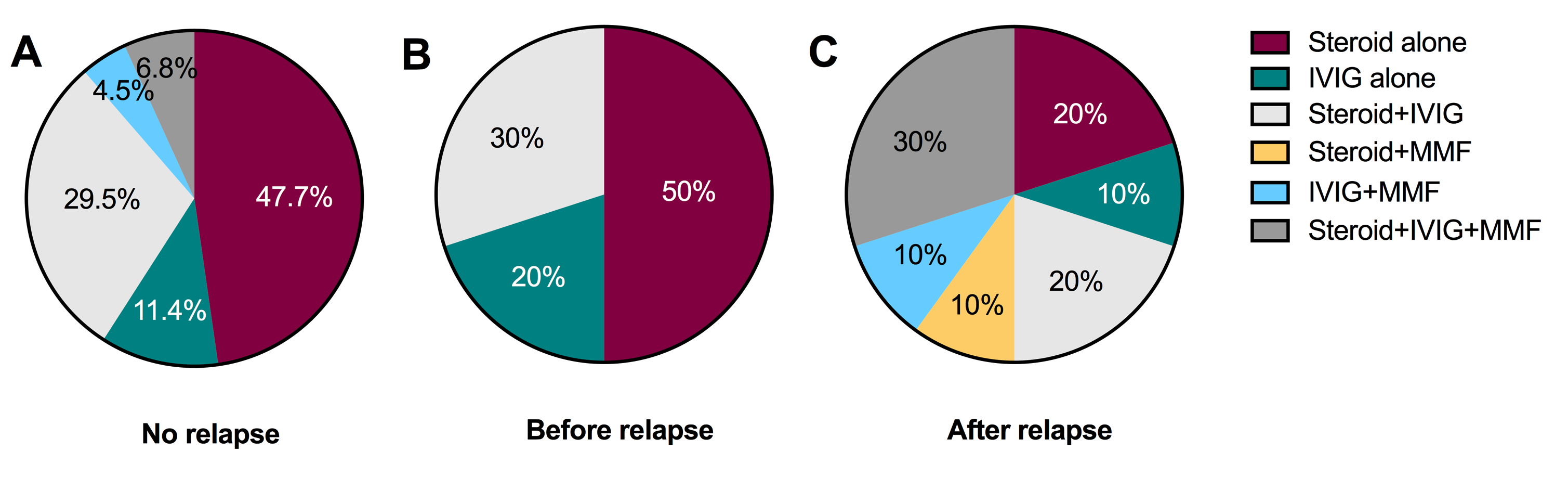

Supplement: Supplementary file 2 [file Image_2.tiff]
