# Supplementary material for: Positive LGI1 Antibodies in CSF and Relapse Relate to Worse Outcome in Anti-LGI1 Encephalitis
Source: Front Immunol. 2021 Dec 17;12:772096. doi: 10.3389/fimmu.2021.772096 (PMC8718904; doi:10.3389/fimmu.2021.772096)
Supplement: Supplementary file 3 [file Table_1.pdf]

Supplemental Table 1. The Barthel Index used to evaluate the patients' abilities of daily living with a full score of 100.

Item		Score
Feeding	Independent	10
	Needs help	5
	Unable	0
Bathing	Independent	5
	Unable	0
Grooming	Independent	5
	Unable	0
Dressing	Independent	10
	Needs help	5
	Unable	0
Bowel control	Continent	10
	Occasional accident	5
	Incontinent (or needs to be given enemas)	0
Bladder control	Continent	10
	Occasional accident	5
	Incontinent (catheterized, unable to manage alone)	0
Toilet use	Independent	10
	Needs help	5
	Unable	0
Transfers (bed to chair and back)	Independent	15
	Needs minor help (verbal or physical)	10
	Needs major help (1-2 people, physical, can sit)	5
	Unable	0
Mobility on level surfaces	Independent (but may use any aid, e.g. stick) >50 yards	15
	Walks with help of one person (verbal or physical) >50 yards	10
	Wheelchair independent, including corners, >50 yards	5
	Immobile or <50 yards	0
Stairs	Independent	10
	Needs help (verbal, physical, carrying aid)	5
	Unable	0
