# Supplementary material for: Positive LGI1 Antibodies in CSF and Relapse Relate to Worse Outcome in Anti-LGI1 Encephalitis
Source: Front Immunol. 2021 Dec 17;12:772096. doi: 10.3389/fimmu.2021.772096 (PMC8718904; doi:10.3389/fimmu.2021.772096)
Supplement: Supplementary file 4 [file Table_2.pdf]

Supplemental Table 2. The Modified Ranking Scale

Symptoms	Score
No symptoms at all	0
No significant disability despite symptoms; able to carry out all usual duties and activities	1
Slight disability; unable to carry out all previous activities, but able to look after own affairs without assistance	2
Moderate disability; requiring some help, but able to walk without assistance	3
Moderately severe disability; unable to walk and attend to bodily needs without assistance	4
Severe disability; bedridden, incontinent and requiring constant nursing care and attention	5
Dead	6
