# Supplementary material for: Positive LGI1 Antibodies in CSF and Relapse Relate to Worse Outcome in Anti-LGI1 Encephalitis
Source: Front Immunol. 2021 Dec 17;12:772096. doi: 10.3389/fimmu.2021.772096 (PMC8718904; doi:10.3389/fimmu.2021.772096)
Supplement: Supplementary file 5 [file Table_3.pdf]

Supplemental Table 3. Comparison of clinical characteristics between patients with positive and negative CSF LGI1 antibodies#

	CSF LGI1 antibody positive (N=42)	CSF LGI1 antibody negative (N=23)	P value
**Male/Female**	24/18	14/11	0.961
**Age at onset, y, mean±SD**	61.2±12.5	53.6±15.0	**0.034***
**Symptom at diagnosis, n (%)**			
**Seizure**	37 (88.1%)	21 (91.3%)	1.0
**Cognitive deficit**	33 (78.6%)	12 (52.2%)	**0.027***
**Psychosis**	14 (34.1%)	5 (21.7%)	0.297
**Consciousness disorder**	7 (16.7%)	2 (8.7%)	0.474
**Speech disorder**	1 (2.4%)	3 (13.0%)	0.118
**Personality change**	6 (19.4%)	7 (30.4%)	0.171
**Sleep disorder**	2 (4.8%)	2 (8.7%)	0.61
**Autonomic dysfunction**	4 (9.5%)	2 (8.7%)	1.0
**Limb weakness**	3 (7.1%)	3 (13.0%)	0.657
**Hyponatremia, n (%)**	21 (50.0%)	7 (30.4%)	0.128
**CSF findings, n (%)**			
**Cell count > 5 cells/uL**	4 (10.0%)	5 (22.7%)	0.259
**Protein > 0.45 g/L**	13 (33.3%)	4 (18.2%)	0.205
**Positive OB**	7 (18.9%)	0 (0.0%)	0.082
**EEG, n (%)**			
**Normal**	7 (16.7%)	5 (21.7%)	
**Epileptic discharge**	9 (21.4%)	10 (43.5%)	
**Slow wave**	6 (14.3%)	1 (4.3%)	0.345
**MRI, n (%)**			
**Normal**	21 (50.0%)	13 (56.6%)	
**Unilateral hippocampal lesion**	12 (28.6%)	3 (13.0%)	
**Bilateral hippocampal lesions**	9 (21.4%)	7 (30.4%)	0.457
**Tumor, n (%)**	3 (7.1%)	3 (13.0%)	0.332
**Initial treatment, n (%)**			
**Corticosteroid**	33 (78.6%)	18 (81.8%)	1.0
**IVIG**	20 (47.6%)	10 (43.5%)	0.683
**MMF**	4 (9.5%)	2 (8.7%)	1.0
**Antiepileptic drugs**	35 (92.1%)	21 (95.5%)	1.0
**Residual symptoms, n (%)**			
**Cognitive impairment**	18 (42.9%)	10 (43.5%)	0.58
**Epilepsy**	9 (21.4%)	3 (13.0%)	0.32
**Personality change**	4 (9.5%)	7 (30.4%)	0.168
**BI upon admission, mean±SD**	76.9±18.4	87.0±17.4	**0.036***
**BI at discharge, mean±SD**	78.8±23.6	90.0±13.1	**0.016***
**Relapse, n (%)**	8 (23.5%)	2 (9.1%)	0.285

**mRS upon admission, median (IQR)**	2 (1~3)	1 (1~1)	**0.003****
**mRS at 12 months, median (IQR)**	1 (0~2)	0 (0~1)	**0.023***

Abbreviations: SD, standard deviation; CSF, cerebrospinal fluid; OB, oligoclonal band; EEG, electroencephalography; MRI, magnetic resonance imaging; IVIG: intravenous immunoglobins; MMF, mycophenolate mofetil; BI, Barthel Index; mRS, modified Ranking scale; IQR, interquartile range. Bold values indicate statistical significance. *P<0.05; **P<0.01. # Two patients were excluded for analysis due to incomplete data.
