# Supplementary material for: Positive LGI1 Antibodies in CSF and Relapse Relate to Worse Outcome in Anti-LGI1 Encephalitis
Source: Front Immunol. 2021 Dec 17;12:772096. doi: 10.3389/fimmu.2021.772096 (PMC8718904; doi:10.3389/fimmu.2021.772096)
Supplement: Supplementary file 6 [file Table_4.pdf]

Supplemental Table 4. Comparison between relapsing and non-relapsing patients

	Non-Relapsing (N=47)	Relapsing (N=10)	P value
**Male/Female**	25/22	5/5	0.564
**Age at onset, y, mean±SD**	56.5±14.2	65.9±12.3	0.056
**Symptom at diagnosis, n (%)**			
**Seizure**	42 (89.4%)	9 (90%)	1
**Memory deficit**	34 (72.3%)	7 (70.0%)	0.579
**Psychosis**	14 (30.4%)	2 (20.0%)	0.406
**Consciousness disorder**	5 (10.6%)	3 (30.0%)	0.137
**Speech disorder**	3 (6.4%)	0 (0.0%)	1
**Sleep disorder**	3 (6.4%)	0 (0.0%)	1
**Autonomic dysfunction**	5 (10.6%)	0 (0.0%)	0.366
**Limb weakness**	4 (8.5%)	0 (0.0%)	1
**Hyponatremia, n (%)**	21 (44.7%)	4 (40%)	0.536
**CSF findings, n (%)**			
**Cell count > 5 cells/uL**	7 (15.6%)	2 (22.2%)	0.469
**Protein > 0.45 g/L**	12 (27.3%)	1 (11.1%)	0.287
**Positive OB**	4 (10.0%)	1 (12.5%)	0.616
**LGI1 antibody, n (%)**			
**CSF positive**	26 (56.5%)	8 (80.0%)	0.154
**Serum positive**	47 (100%)	10 (100%)	1
**EEG, n (%)**			
**Epileptic discharge**	17 (53.1%)	1 (50%)	1
**Slow wave**	5 (15.6%)	1 (50%)	0.477
**MRI, n (%)**			
**Normal**	23 (49.0%)	7 (70.0%)	
**Unilateral hippocampal lesion**	12 (25.5%)	1 (10.0%)	
**Bilateral hippocampal lesions**	12 (25.5%)	2 (20.0%)	0.512
**Tumor, n (%)**	4 (9.1%)	2 (20%)	0.456
**Treatment, n (%)**			
**Corticosteroid**	37 (80.4%)	8 (80.0%)	0.637
**IVIG**	23 (50.0%)	5 (50.0%)	1
**MMF**	5 (10.9%)	0 (0.0%)	0.573
**Antiepileptic drugs**	40 (95.2%)	9 (90%)	0.481
**Residual symptom, n (%)**			
**Cognition impairment**	21 (44.7%)	7 (70.0%)	0.148
**Epilepsy**	6 (12.8%)	6 (60.0%)	**0.002****
**Personality change**	9 (19.1%)	2 (20.0%)	1
**BI upon admission, mean±SD**	81.2±19.0	74±20.1	0.286
**BI at discharge, mean±SD**	85.2±17.6	72.5±24.0	0.057
**mRS upon admission, median (IQR)**	1 (1~3)	1 (1~3)	0.954
**mRS at 12 months, median (IQR)**	0 (0~1.25)	1 (1~1.75)	0.15

Abbreviations: SD, standard deviation; CSF, cerebrospinal fluid; OB, oligoclonal band; EEG, electroencephalography; MRI, magnetic resonance imaging; IVIG: intravenous immunoglobins; MMF, mycophenolate mofetil; BI, Barthel Index; mRS, modified Ranking scale; IQR, interquartile range. Bold value indicates statistical significance. *P<0.05; **P<0.01.
